# Supplementary material for: Tunisian Multicenter Study on the Prevalence of Colistin Resistance in Clinical Isolates of Gram Negative Bacilli: Emergence of Escherichia coli Harbouring the mcr-1 Gene
Source: Antibiotics (Basel). 2022 Oct 11;11(10):1390. doi: 10.3390/antibiotics11101390 (PMC9598684; doi:10.3390/antibiotics11101390)
Supplement: Supplementary file 1 [file antibiotics-11-01390-s001.zip › antibiotics-1862124-supplementary.pdf]

Table S1. Oligonucleotides used in this study.

Gene	Primer[a]	Sequence (5′–3′)[c]	Product Size (bp)[b]	Reference
ACC-1 and ACC-2	ACC-F	CACCTCCAGCGACTTGTTAC	346	[16]
	ACC-R	GTTAGCCAGCATCACGATCC		
FOX-1 à FOX-5	FOX-F	CTACAGTGCGGGTGGTTT	162	
	FOX-R	CTATTTGCGGCCAGGTGA		
MOX-1, MOX-2, CMY-1, CMY-8 to CMY-11 and CMY-19	MOX-F	GCAACAACGACAATCCATCCT	895	
	MOX-R	GGGATAGGCGTAACTCTCCCAA		
DHA-1 and DHA-2	DHA-F	TGATGGCACAGCAGGATATTC	997	
	DHA-R	GCTTTGACTCTTTCGGTATTCG		
LAT-1 to LAT-3, BIL-1, CMY-2 to CMY-7, CMY-12 to CMY-18 and CMY-21 to CMY-23	CIT-F	CGAAGAGGCAATGACCAGAC	538	
	CIT-R	ACGGACAGGGTTAGGATAGY		
ACT-1 and MIR-1	EBC-F	CGGTAAAGCCGATGTTGCG	683	
	EBC-R	AGCCTAACCCCTGATACA		
mcr-1[*]	CLR-F	C GGTCAGTCCGTTTGTTC	309	[4]
	CLR-R	CTTGGTCGGTCTGTAGGG		
mcr-2	Mcr-2-F	CAAGTGTGTTGGTCGCAGTT	715	
	Mcr-2-R	TCTAGCCCGACAAGCATACC		
mcr-3	Mcr-3-F	AAATAAAAATTGTTCCGCTTATG	929	
	Mcr-3-R	AATGGAGATCCCCGTTTTT		
mcr-4	Mcr-4-F	TCACTTTCATCACTGCGTTG	1116	
	Mcr-4-R	TTGGTCCATGACTACCAATG		
mcr-5	Mcr-5-F	ATGCGGTTGTCTGCATTTATC	1644	
	Mcr-5-R	TCATTGTGGTTGTCCTTTTCTG		
mcr-6	mcr-6-F	AGCTATGTCAATCCCGTGAT	252	[14]
	mcr-6-R	ATTGGCTAGGTTGTCAATC		
mcr-7	mcr-7-F	GCCCTTCTTTTCGTTGTT	551	
	mcr-7-R	GGTTGGTCTCTTTCTCGT		
mcr-8	mcr-8-F	TCAACAATTCTACAAAGCGTG	856	
	mcr-8-R	AATGCTGCGCGAATGAAG		
mcr-9	mcr-9-F	TTCCCTTTGTTCTGGTTG	1011	
	mcr-9-R	GCAGGTAATAAGTCGGTC		
*bla*IMP	IMP-F	GGAATAGAGTGGCTTAAYTCTC	232	[17]
	IMP-R	GGTTTAAYAAAACAACCACC		
*bla*NDM	NDM-F	GGTTTGGCGATCTGGTTTTC	621	
	NDM-R	CGGAATGGCTCATCACGATC		
*bla*KPC	KPC-F	CGTCTAGTTCTGCTGTCTTG	798	
	KPC-R	CTTGTCATCCTTGTTAGGCG		
*bla*OXA-48	OXA-48-F	GCGTGGTTAAGGATGAACAC	438	
	OXA-48-R	CATCAAGTTCAACCCAACCG		
*bla*GES	GES-F	AGTCGGCTAGACCGGAAAG	399	
	GES-R	TTTGTCCGTGCTCAGGAT		
*bla*TEM variants including *bla*TEM-1 and *bla*TEM-2	MultiTSO-T-F	CATTTCCGTGTCGCCCTTATTC	800	[16]
	MultiTSO-T-R	CGTTCATCCATAGTTGCCTGAC		
*bla*SHV variants including *bla*SHV-1	MultiTSO-S-F	AGCCGCTTGAGCAAATTAAAC	713	
	MultiTSO-S-R	ATCCCGCAGATAAATCACCAC		

*bla*OXA-1, *bla*OXA-4 and *bla*OXA-30	MultiTSO-O-F	GGCACCAGATTCAACTTTCAAG	564	
	MultiTSO-O-R	GACCCCAAGTTTCCTGTAAGTG		
variants of *bla*CTX-M group 1 including *bla*CTX-M-1, *bla*CTX-M-3 and *bla*CTX-M-15	CTXMGp1-F	TTAGGAARTGTGCCGCTGYAb	688	
	CTXMGp1-2-R	CGATATCGTTGGTGGTRCCATb		
variants of *bla*CTX-M group 2 including *bla*CTX-M-2	CTXMGp2-F	CGTTAACGGCACGATGAC	404	
	CTXMGp1-2-R	CGATATCGTTGGTGGTRCCATb		
variants of *bla*CTX-M group 9 including *bla*CTX-M-9 and *bla*CTX-M-14	CTXMGp9-F	TCAAGCCTGCCGATCTGGT	561	
	CTXMGp9-R	TGATTCTCGCCGCTGAAG		
*bla*CTX-M-8, *bla*CTX-M-25, *bla*CTX-M-26 and *bla*CTX-M-39 to *bla*CTX-M-41	CTX-Mg8/25-F	AACRCRCAGACGCTCTACb	326	
	CTX-Mg8/25-R	TCGAGCCGGAASGTGTYATb		
chuA	chuA.1b	ATGGTACCGGACGAACCAAC	288	[20]
	chuA.2	TGCCGCCAGTACCAAAGACA		
yjaA	yjaA.1b	CAAACGTGAAGTGTCAGGAG	211	
	yjaA.2b	AATGCGTTCCTCAACCTGTG		
TspE4C2	TspE4C2.1b	CACTATTCGTAAGGTCATCC	152	
	TspE4C2.2b	AGTTTATCGCTGCGGGTCGC		
arpA	AceK.f	AACGCTATTCGCCAGCTTGC	400	
	ArpA1.r	TCTCCCCATACCGTACGCTA		
arpA	ArpAgpE.f	GATTCCATCTTGTCAAAATATGCC	301	
	ArpAgpE.r	GAAAAGAAAAAGAATTCCCAAGAG		
trpA	trpAgpC.1	AGTTTTATGCCCAGTGCGAG	219	
	trpAgpC.2	TCTGCGCCGGTCACGCCC		
trpA (contrôle interne)	trpBA.f	CGGCGATAAAGACATCTTCAC	489	
	trpBA.r	GCAACGCGGCCTGGCGGAAG		
int1	int1-F	GGG TCA AGG ATC TGG ATT TCG	438	[19]
	int1-R	ACA TGC GTG TAA ATC ATC GTC G		
int2	Int2-F	CAC GGA TAT GCG ACA AAA AGG T	788	
	Int2-R	GTA GCA AAC GAG TGA CGA AAT G		
int3	Int3-F	GCC TCC GGC AGC GAC TTT CAG	500	
	Int3-R	ACG GAT CTG CCA AAC CTG ACT		
	MCR1-END-F	GTGCGAACATCAGTCCTTGA		[6]
	PAP2-INT-R	CGCAACCAGCAAGTAGATCA		
ISApl1	ISApl1-mcr-F	TGGACATTGGGAAGCCGATA		[15]
	ISApl1-mcr-R	GCCA CAAGAACAAACGGACT		
IncB/O	B/O-F	GCGGTCCGGAAAGCCAGAAAAC	159	[18]
	B/O-R	TCTGCGTTCCGCCAAGTTCGA		
IncFIC	FIC-F	GTGAACTGGCAGATGAGGAAGG	262	
	FIC-R	TTCTCCTCGTCGCCAAACTAGAT		
IncA/C	A/C-F	GAGAACCAAAGACAAAGACCTGGA	465	
	A/C-R	ACGACAAACCTGAATTGCCTCCTT		

IncP	P-F	CTATGGCCCTGCAAACGCGCCA GAAA	534
	P-R	TCACGCGCCAGGGCGCAGCC	
IncT	T-F	TTGGCCTGTTTGTGCCTAAAC CAT	750
	T-R	CGTTGATTACACTTAGCTTTGGA C	
IncK/B	K/B-F	GCGGTCCGGAAAGCCAGAAAAC	160
	K/B-R	TCTTTCACGAGCCCGCCAAA	
IncW	W-F	CCTAAGAACAACAAAGCCCCCG	242
	W R	GGTGCGCGGCATAGAACCGT	
IncFIIA	FIIA-F	CTGTCGTAAGCTGATGGC	270
	FIIA-R	CTCTGCCACAAACTTCAGC	
IncFIA	FIA F	CCATGCTGGTTCTAGAGAAGGTG	462
	FIA R	GTATATCCTTACTGGCTTCCGCAG	
IncFIB	FIB-F	GGAGTTCTGACACACGATTTTCT G	702
	FIB R	CTCCCGTCGCTTCAGGGCATT	
IncY	Y F	AATTCAAACAACACTGTGCAGCC TG	765
	Y-R	GCGAGAATGGACGATTACAAAA CTTT	
IncI1	I1-F	CGAAAGCCGGACGGCAGAA	139
	I1-R	TCGTCGTTCCGCCAAGTTCGT	
Frep	Frep F	TGATCGTTTAAGGAATTTTG	270
	Frep-R	GAAGATCAGTCACACCATCC	
IncX	X-F	AACCTTAGAGGCTATTTAAGTTG CTGAT	376
	X-R	TGAGAGTCAATTTTTATCTCATGT TTTAGC	
IncHI1	HI1-F	GGAGCGATGGATTACTTCAGTAC	471
	HI1-R	TGCCGTTTCACCTCGTGAGTA	
IncN	N-F	GTCTAACGAGCTTACCGAAG	559
	N-R	GTTTCAACTCTG CCAAGTTC	
IncHI2	HI2-F	TTTCTCCTGAGTCACCTGTTAACAC	644
	HI2 R	GGCTCACTACCGTTGTCATCCT	
IncL/M	L/M-F	GGATGAAAACTATCAGCATCTGA AG	785
	L/M-R	CTGCAGGGGCGATTCTTTAGG	

[a] F, sense primer; R, antisense primer. [b] Nucleotide numbering begins at the initiation codons of genes. [c] D = A or G or T; Y = C or T.
